# Modeling the Repertoire of True Tumor-Specific MHC I Epitopes in a Human Tumor

**DOI:** 10.1371/journal.pone.0006094

**Published:** 2009-07-10

**Authors:** Nisheeth Srivastava, Pramod K. Srivastava

**Affiliations:** 1 Department of Computer Science, University of Minnesota, Minneapolis, Minnesota, United States of America; 2 Center for Immunotherapy of Cancer & Infectious Diseases, University of Connecticut School of Medicine, Farmington, Connecticut, United States of America; Harvard Institute of Medicine, United States of America

## Abstract

DNA replication has a finite measurable error rate, net of repair, in all cells. Clonal proliferation of cancer cells leads therefore to accumulation of random mutations. A proportion of these mutational events can create new immunogenic epitopes that, if processed and presented by an MHC allele, may be recognized by the adaptive immune system. Here, we use probability theory to analyze the mutational and epitope composition of a tumor mass in successive division cycles and create a double Pölya model for calculating the number of truly tumor-specific MHC I epitopes in a human tumor. We deduce that depending upon tumor size, the degree of genomic instability and the degree of death within a tumor, human tumors have several tens to low hundreds of new, truly tumor-specific epitopes. Parenthetically, cancer stem cells, due to the asymmetry in their proliferative properties, shall harbor significantly fewer mutations, and therefore significantly fewer immunogenic epitopes. As the overwhelming majority of the mutations in cancer cells are unrelated to malignancy, the mutation-generated epitopes shall be specific for each individual tumor, and constitute the antigenic fingerprint of each tumor. These calculations highlight the benefits for personalization of immunotherapy of human cancer, and in view of the substantial pre-existing antigenic repertoire of tumors, emphasize the enormous potential of therapies that modulate the anti-cancer immune response by liberating it from inhibitory influences.

## Introduction

DNA replication is arguably central to life, and it occurs with less than complete fidelity. The imperfection in fidelity leads to a measurable error rate, net of repair, that is an essential and inalienable component of any cell division, bacterial or mammalian, normal or malignant. The estimates of the spontaneous mutation rate vary from 10^−5^ to 10^−9^ per nucleotide per cell cycle, depending upon the experimental system used [1]; much higher rates obtain in case of cells with genetic instability [2], [3] either because of deficient DNA repair [4], or chromosomal instability [5]. The mutations create the substratum for natural selection and origin of species and malignant cancers alike. Prior mathematical models for studying the role of genomic instability in tumorigenesis [6] have relied upon simple compounding models of growth to estimate the total number of mutations in a fully grown tumor.

We demonstrate here that such mutations can be effectively captured using a double Pölya urn scheme, and that doing so allows us the mathematical flexibility to answer important biological questions. Specifically, we address a facet of the mutational repertoire of cancers, that has not received mathematical attention, i.e. the immunological consequences of the mutational burden of tumors. One of us has previously suggested that the mutational burden of tumors must inevitably lead to generation of tumor-specific neo-antigens that must be unique for each individual tumor because of randomness of the mutational process [7]. However, no attempt at quantitative modeling of this important phenomenon has been made by us or others. This is largely due to two factors: the rules of immunological recognition, specifically, the recognition of a complex of MHC I with stretches of amino acids by CD8+ T lymphocytes, are considerably more intricate, and hence less amenable to modeling than the binary rules of mutations. Secondly, these rules have become clear only during the last 10–15 years [8], [9], [10]. The results of our analyses reveal that the tumor-specific repertoire of antigens is vast and individually unique. Indeed, this conclusion was inherent in the earliest experiments that pointed to the specific immunogenicity of tumors (see [11] for review). These early studies, carried out with transplantable but syngeneic tumors showed two distinct phenomena: (i) each tumor could be used to immunize mice (or rats) and the immunized animals were resistant to subsequent tumor challenge with the immunizing tumor; (ii) the tumor reistance was restricted to the tumor that was used to immunize. Mice immunized to one tumor and resistant to it were still sensitive to challenge with another tumor, even if the othe other tumor was of the same histological origin, was induced in the same strain of mice and by the same carcinogen, as the immunizing tumor. A rigorous scrutiny of these phenomena using a large panel of chemically induced tumors [12] still upheld the observations of individually-specific immunogenicity of tumors initially made with smaller numbers of tumors. As argued previously [7], these results could be explained on basis of an antigen repertiore generated by random mutations. Our present results provide a mathematical form to that argument, raise a number of testable questions and predictions and suggest novel avenues of immunotherapy of human cancer.

## Results

### The basic model of mutational burden of a tumor

A very small number of admittedly simplistic assumptions are used to create a basic model into which more realistic components may be incorporated. The assumptions are: (a) A cancer is clonal in origin. (b) The mutation rate in the cancer is invariant through the cancer's lifetime. Genetic instability including repair deficiencies and chromosomal instability, are not modeled in the basic model, but have been incorporated in its variants. (c) Cancer cells die at an invariant rate through the cancer's life time. Selected variations in death rates during a tumor's evolution may be added to the basic model. (d) The mutations are all point mutations, and no reversions occur. This is perhaps the most simplistic of all assumptions. The mutational complexity of tumors including deletions and insertions is fully acknowledged, but not represented in our models. (e) All mutations are ‘equal’ such that no mutation confers a survival advantage or disadvantage to the cell harboring it. This clearly incorrect assumption is made because an overwhelming majority of mutations indeed are ‘equal’ and are incidental to survival or malignant transformation. In light of these simplifications, our model represents a minimal representation of tumor-associated genetic changes.

The classical formulation of Pölya's urn problem can be stated as follows: an urn initially contains *r* red and *b* blue marbles. One marble is chosen randomly from the urn. The marble is then put back into the urn together with *c* more marbles (presumably from a collection stored elsewhere) of the same color. Results computing the probability of the existence of *k* red marbles in the urn after *t* trials are well-known.

If we model the reproduction of each individual base pair in this setting, it is immediately evident that c = 0 in this case, since the size of the genome remains constant. Hence, we get a binomial distribution over the number of mutations in a cell cycle. This may be represented as,
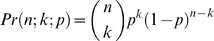
(1)where n is the number of base pairs, *p* is the probability of faulty reproduction of a single base pair, and *k* is the number of mutations in the entire DNA sequence in the daughter cells.

Now, we must derive an updated equation for the change in the number of mutations across cell cycles, where the number of mutations produced in each cycle follow the same generative model as shown in Eqn. (1). In that case, we can compute the probability of the existence of 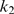 mutations, given the existence of 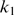 mutations 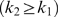
in the previous cell cycle as
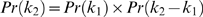
Thus, recursively, it follows that the probability of seeing k mutations in the 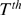 cell cycle will be
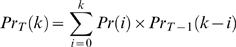
(2)Alternatively, we can derive an analytical expression for 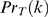 if we assume that mutation of an already mutated base pair is statistically irrelevant. This is a completely justifiable assumption, and allows us to calculate the probability 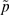 that a single base pair will mutate across a series of *T* cycles as,
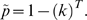
(3)Using 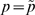 in Eqn (1), we will obtain the probability of the existence of *k* mutations in the 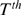 cell cycle as,
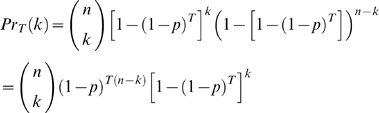
(4)


In light of the calculations described above, starting with a diploid human genome of 6×10^9^ bp, and assuming a conservative spontaneous mutation rate of 5×10^−9^/bp/cell cycle, the average number of mutations generated in each cell cycle is simply the mean of the binomial distribution in Eqn. (1) and is calculated as 30.

**Figure 1:**
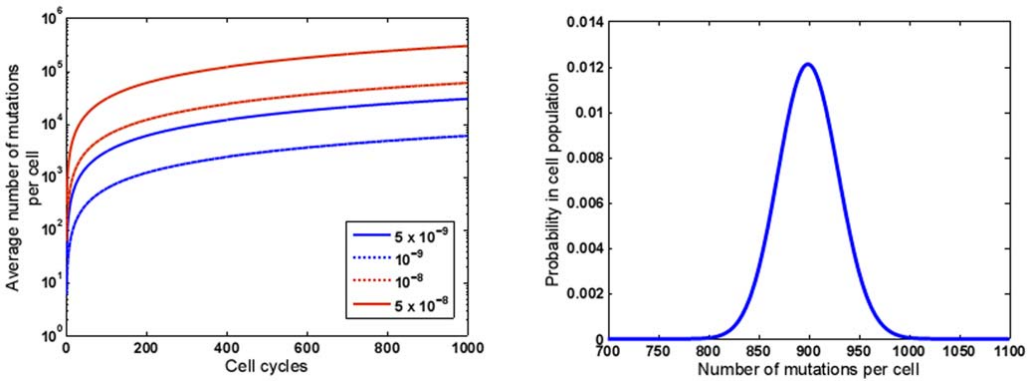
Modeling the numbers of accumulating mutations in dividing cells using a probabilistic model. (a) Prediction of average number of accumulated mutations per diploid human cell as a function of numbers of cell cycles. The model assumes a diploid DNA content of 6×10^9^ bp and a number of possible mutation rates (10^−9^,5×10^−9^,10^−8^,5×10^−8^ per bp per cell cycle) as indicated. (b) The numerical profile of mutations in a clonally derived cell population of approximately 10^9^ cells (after 30 division cycles). A spontaneous mutation rate of 5×10^−9^ is assumed.

Next we consider the average number of mutations per cancer cell in a tumor of size 1 cm^3^ or approximately 10^9^ cells. A cancer cell would have to undergo approximately 30 cycles to arrive at that size. As each daughter cell would retain the mutations that it inherits from its parent, the average cumulative number of mutations per cell at the end of 30 cycles, shall be 900. With increasing number of cycles, and increasing tumor mass, an increasing number of mutations will accumulate linearly per cell Fig. 1(a). The actual distribution of mutational complexity follows the probability density defined in Eqn. (4) and is visualized in Fig. 1(b). As each cycle shall generate random mutations anew, the various cells in the tumor shall not have a homogeneous composition, but shall be mosaics of overlapping compositions. Altogether, this tumor of 10^9^ cells shall harbor ∼9×10^11^ mutations. If we assume that mutations that occur in less than 10% of the total cells in the tumor are undetectable in the laboratory, mutations that occur after the fourth cell cycle will be undetectable. Therefore, the actual number of mutations that we would expect to find in a tumor of size 1 cm^3^ under experimental conditions will be about 1.2×10^11^.

Should one assume the presence of a mutator mutation in the parental cancer cell that enhances the mutation rate one hundred fold, one similarly arrives at a number of 90,000 mutations per cell by the time the tumor achieves a size of 10^9^ cells. Under these conditions, one out of every 60,000 bp shall have undergone a mutation.

### Death rate and the number of mutations

**Figure 2:**
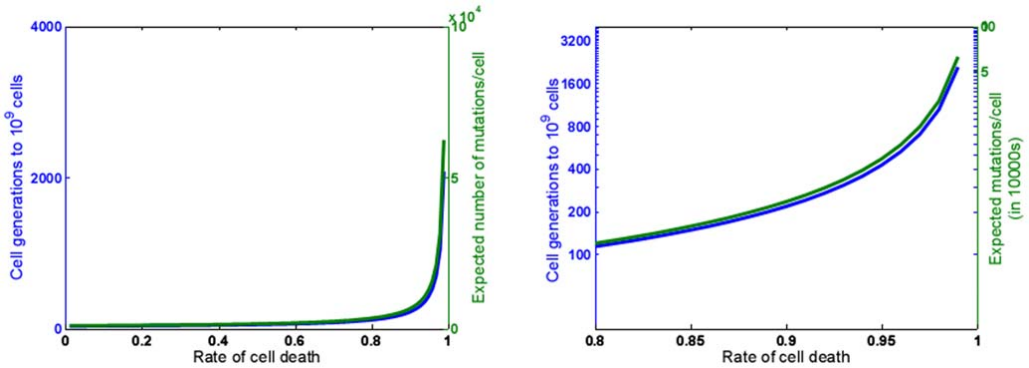
Modeling the numbers of accumulating mutations in dividing cells as a function of rates of cell death using a probabilistic model. Rate of cell death is defined as the fraction of cells dying in each generation. As an example, if a single cell divides into two, and only 1.6 of these two cells survive, the death rate is denoted as 0.4. (a) Number of cell cycles required for a tumor to grow from a single cell to 10^9^ cells (≈1 cm^3^) as a function of rates of cell death. The higher the death rates, the more times the cells have to divide to create the same size of tumor. Note on the right vertical axis, that the number of accumulating mutations per cell also rises with the number of cell divisions undergone; the numbers are plotted with an assumed mutation rate of 5×10^−9^ per bp per cell division cycle. (b) The region of (a) denoting death rates between 0.8 and 1 is magnified; death rates between 0.8 and 1 represent the most realistic scenario for a tumor growing in vivo. Note that the vertical axis is plotted on a logarithmic scale.

In the scenario envisioned in the previous section, thirty cycles shall be achieved within a month, assuming no stasis or cell death. While situations of invariant cell death rates must arise occasionally in course of evolution of human tumors, it is an unlikely scenario during genesis of a tumor. A new developing tumor, or a newly metastatic lesion undergoes successive cycles of vigorous expansion and cell death depending upon whether or not it is vascularized, the extent of immunological attack it encounters, in addition to other inchoate factors. This scenario cannot be modeled with any degree of accuracy. One can however consider an invariant and uniform death rate 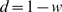, where *w* is the fractional growth rate of the tumor cell population. We can now calculate the number of cell cycles required for the tumor to attain a certain size N using,
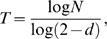
(5)and use this value of T in Eqn. (4) to estimate the number of mutations in the cell population. The higher the death rate, the more cycles a cell would have undergone before achieving a certain size Fig. 2(a). As more cycles inevitably involve more mutations, a tumor with a higher death rate would have a larger number and larger complexity of mutations than a tumor with a lower death rate Fig. 2(b).

Revising our estimates to account for a steady and random death rate, we work with a reasonable assumption that it takes 300 cycles (about ten months) for a tumor to grow from a cell to a size of 1 cm^3^ which is ∼10^9^ cells. In this case, the expected number of mutations in the grown tumor will multiply ten-fold from the previous figure of 900 mutations to 9000 mutations per cell. Calculating the number of experimentally observable mutations will follow a somewhat different route in this case. We will have to assume that mutations that occur after the tumor has reached 10% of its final size will not be detectable. We will now have 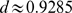, which implies that mutations that occur after 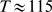 will not be detectable. Thus, we get an expected value of 3450 experimentally detectable mutations per cell in a tumor of about 1 cm^3^ or ∼10^9^ cells, assuming a mutation rate of 5×10^−9^/bp/cell cycle and a modest death rate. This calculation is valid for tumors of the same size; number of mutations will be proportionately higher in larger tumors, or tumors with higher rates of mutations and higher rates of cell death.

### From mutational content to definition of the tumor immunome

Starting with the deduction that a tumor of 10^9^ cells harbors an average of 9000 mutations per cell (over 300 cell cycles), and with the assumption that mutations are distributed randomly between the coding and non-coding segments of the genome, one can calculate the number of mutations in the coding genome, at 1.5% or 135 per cell. Calculating that one third of these mutations shall fall on each of the three positions of a triplet codon, and further that mutations in the first and second positions shall be productive, and those in the third position, silent, one arrives at a number of 90 alterations in the coding sequences of this tumor cell. How many new antigenic epitopes do these alterations create?

In order to model this, we have chosen to focus on the epitopes that can be potentially processed and charged onto MHC I molecules and potentially recognized by CD8+ T lymphocytes. While other aspects of the immune system play important roles in immunological resistance to cancer, the MHC I -restricted, antigen-specific response plays a central role. In order to determine the number of MHC I epitopes that shall be generated by the 90 productive mutations per cell, we wanted to identify the possible number of sites in the coding genome which were one amino acid ‘short’ of a consensus HLA I motif, and which therefore could be converted into a perfect motif by a single point mutation. However, no such super-motif exists. We have approached the problem by narrowing our calculations to HLA A2, one of the more common allele and one for which a well-defined motif - (a 9-mer peptide with small and aliphatic residues ATSVLIMQ in the B pocket and aliphatic and small hydrophobic residues ALIVMQ in the F pocket) -exists [9].

**Figure 3:**
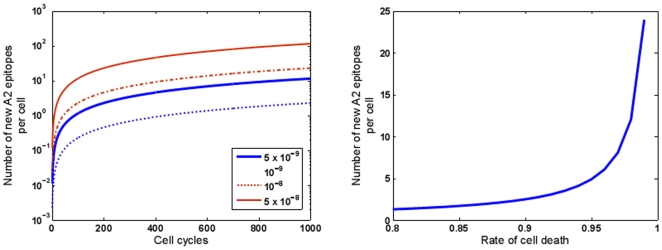
Modeling the numbers of accumulating tumor-specific HLA A2-restricted neo-epitopes in dividing human cancer cells using a probabilistic model. (a) Prediction of average number of accumulated A2-restricted epitopes per cell as a function of numbers of cell cycles. The model assumes a diploid DNA content of 6×10^9^ bp and a number of possible mutation rates (10^−9^,5×10^−9^,10^−8^,5×10^−8^ per bp per cell cycle) as indicated. The higher mutation rates are more representative of human cancers. (b) The expected numbers of A2-restricted tumor-specific neo-epitopes in tumors (≈1 cm^3^ size) with varying inherent rates of cell death. The higher the death rate, the higher the number of cell cycles required for a tumor to grow to a certain size (as shown in Fig. 2), and the higher the number of tumor-specific neo-epitopes. This figure assumes a mutation rate of 5×10^−9^ per bp per cell division in the tumor.

To compute the number of HLA A2 alleles that will arise as a consequence of random mutations, we follow a simple line of probabilistic argument, outlined below,

1. We have calculated that the average number of productive mutations in the coding region of the genome of a tumor of a cell mass of 10^9^ cells is about 90/cell.
2. The coding region of the genome is taken to comprise of about 1.5% of the total sequence ∼9×10^7^ base pairs, translated to ∼3×10^7^ [13].
3. Since 9-mers can overlap, the total number of possible 9-mers can also be taken to be ∼3×10^7^.
4. Recall that A2 motifs are characterized by the combined presence of one of 8 residues in the B pocket and one of 6 residues in the F pocket. To compute the probable number of HLA A2 motifs in the coding region, we therefore use a combinatorial construction viz.,
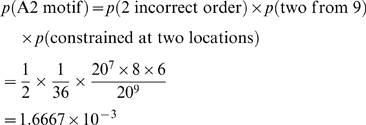
The total number of A2 motifs in the coding region of the genome will then be,
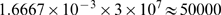

5. We now must find the number of motifs that are one mutation away from being recognized as A2 motifs. For a motif to be one mutation away from being recognized as A2, it must already have a compatible residue in either the B or the F pocket and an incompatible one in the other. Therefore, to find the probable number of motifs one mutation away from A2, we use a combinatorial construction as above to compute the probability of each of these two exclusive cases individually and then add them. This is calculated as,
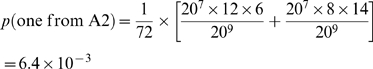
The total number of epitopes one mutation away from A2 will then simply be,
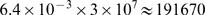

6. We know, from previous calculations the number of active mutations in the coding region of the genome. Operating under the assumption that these mutations occur randomly, we can compute the average value for the number of such ‘false positive’ incidences in the genome. This comes out to be,
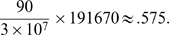
Thus, we conclude that the total number of 9-mer motifs that can mutate and be recognized as HLA A2 motifs is of the order of 0.6 epitopes per cell, in a tumor that has arisen from 300 division cycles. (Parenthetically, these calculations assume that the mutations are neutral with respect to their effects on proteasome cleavage and transport of peptides through transporters associated with antigen processing.) This number will clearly increase as the tumor undergoes more cell cycles Fig. 3(a). With a total of 6 MHC I alleles, and assuming that the frequency of other alleles is similar to A2, there may be up to 3.6 total new tumor-specific MHC I epitopes per tumor cell at this stage. The number of such epitopes shall clearly increase in a tumor that is larger, or that has a higher mutation rate as a result of genomic instability, or one that has undergone a larger number of cycles for any reason,including a higher death rate Fig. 3(b). Thus, in the not uncommon scenario of a tumor with a hundred fold higher mutation rate, one may expect 360 new tumor-specific MHC I epitopes per tumor cell. Clearly, the actual number of new epitopes may be anywhere between 3.6 and 360 per cell depending upon the mutation rate. While this manuscript was under preparation for submission, we became aware of the study by Segal et al. [14] where the authors have actually analyzed in silico the number of possible tumor-specific HLA A201 epitopes based on known partial sequences of tumor transcripts; they calculate individual breast and colon cancers to have between 7 and 10 new epitopes. These numbers are clearly consistent with our theoretical predictions.

## Discussion

### Summary of results

Our study models the size of the repertoire of tumor-specific MHC I epitopes in a tumor, starting from first principles of genetics. We hasten to emphasize that the modeling here is restricted to truly tumor-specific epitopes, that may not be present in normal tissues. An extensive database that documents the repertoire of MHC I epitopes that are shared between normal tissues and tumors (such as differentiation antigens, cancer testes antigens etc) exists [15], [16], and is not the subject of this analysis. Our analyses show that (a) each cell of a relatively small human tumor of 1 cm^3^ harbors approximately 900 individual mutations, assuming a spontaneous mutation rate of 5×10^−9^/bp/cell cycle. If one factors in the presence of mutator mutations, or other mechanisms of genetic instability, a proportionately higher number of mutations is obtained. Corresponding numbers of mutations can be derived for other mutation rates, higher and lower. Accepting that a mutation must exist in at least 10% of the cells in order to be detectable by DNA amplification methods, one would detect 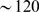 mutations per cell in this tumor at the basic mutation rate. (b) If the modeling takes into account the fact that a significant proportion of cancer cells die even as the cancer progresses, the number of mutations in a tumor varies directly with the death rate; the higher the death rate during the tumor's progression, the higher the number of mutations; (c) Translation of the spontaneous mutations at the basic rate of 5×10^−9^/bp/cell cycle to the changes in amino acid composition of the proteome suggests that a human tumor of 1 cm^3^ shall harbor 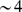 new tumor-specific epitopes per tumor cell. In a larger tumor, and in tumors with higher mutation rates due to genetic instability, or tumors with certain death rates, a substantially higher number of new MHC I epitopes is generated, such that a clinically detectable tumor may harbor hundreds of tumor-specific epitopes.

This model has a number of limitations. The number of all potential A2 epitopes calculated is based on the assumption that each of the possible twenty amino acids can occupy any position in a protein. This is clearly not so, and corrections for this factor shall alter the final numbers to a minor degree. Secondly, not all potential epitopes may be generated due to constraints in processing, the half life of proteins and other factors [17], [18]. Hence, the number of actual as opposed to potential epitopes may be as low as 10% of the modeled number. Conversely, the model only considers point mutations, and thus ignores considerable sources of additional genetic and hence immunogenic alterations. These limitations, in either direction, should be borne in mind in interpreting the physiological consequences of our model.

### Comparison with previous results

Tomlinson et al. [6] have estimated 1250 mutations per adenocarcinoma cell (in a cancer that has grown over 1000 cell cycles), assuming a mutation rate of 5×10^−9^ per bp per cell cycle. The results of our calculations lead us to qualitatively similar results. Our estimates are also generally consistent with the number of 10,000 mutations per cell arrived at experimentally by Stoler et al. [19] They are also concordant with the range of frequency of “passenger” somatic mutations observed by Greenman et al. in an array of cancer genomes [20]. Our model operates at the level of probabilities of mutations at the level of single nucleotides, as opposed to the geometric series used by Tomlinson et al. The probabilistic approach, while harder to implement, allows resolution of questions not addressable by the geometric progression approach. The modeling of the size of the epitope repertoire, as performed here, is one such question. No previous study has modeled the number of new tumor-specific epitopes generated as a result of tumor progression, and hence such a comparison is not possible. However, a comment regarding the estimates regarding the total number of A2 epitopes present in normal proteome is instructive. We calculate as 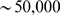 the total number of such epitopes. Intestingly, Assarson et al [17]. calculate that the number of A2 epitopes in a 100 amino acid stretch of the vaccinia virus genome to be about 2.5. If the total human proteome consisting of 10^7^ amino acids were to follow similar rules, it may be expected to contain 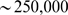 A2 epitopes by their calculation. The modeled number of A2 epitopes per our calculations is about 20 percent of that calculated by Assarson et al., and thus significantly, but not qualitatively different. The most pertinent calculations for us are those made by Segal et al [14]; these authors applied in silico-based epitope prediction algorithms on 1152 peptides containing missense mutations in breast and colorectal cancers and calculated that individual cancers have between 7 and 10 new tumor-specific HLA 0201 epitopes. These numbers are quite close to those arrived at in our calculations.

### Testable predictions on molecular genetics of human tumors

A number of predictions have been made regarding the numbers of mutations in human cancers. While the human genome of two human individuals has been sequenced, the complete sequence of a human, or a murine tumor genome remains to be determined. Considering the sliding costs of sequencing, it is now well within the realm of possibility that one or more tumor genomes shall be sequenced in short order. Such an analysis, preferably carried out along with the non-tumor genome of the same individual, shall be enormously informative with respect to the models generated here and in other studies. It is worth re-emphasizing here that our present model only considers point mutations, and not the other more significant forms of genetic modifications including chromosomal instability, deletions etc. As such, our models present a minimal picture of the genetic changes associated with carcinogenesis. The other aspect that is implicit in our model is that of uniqueness of the genetic signature of each individual cancer. As the mutations are assumed to be random, the non-malignancy associated mutations, and these are presumably the most of them, would be unique to each tumor. This prediction shall also be put to test by the sequencing efforts.

### Implications for immune responses to cancers

Our model has several novel implications. It suggests that a growing tumor is not immunologically recognizable because at the very early stages, it does not have any truly tumor-specific immunogenic epitopes! In the simplest scenario in our model, a tumor without genetic instability and without significant rates of cell death may easily grow to a size of 1 cm^3^ and may have only a single immunogenic epitope, if that, at that stage. This provides a perfect mechanism for tumors to grow un-detected under the immunological radar. As they grow larger, they of course shall become more immunogenic, and hence more visible. Very interestingly, Gatenby et al [21]. have reached a similar conclusion purely on information-theoretic grounds. They conclude that the Fisher information of a tumor is very low in the early stages of its growth, resulting in an error of at least 30% in the best possible estimate of its time of origin. We find it gratifying that our probabilistic treatment of nucleotide mutation predicts the same result. With the time that it takes tumors to achieve larger sizes, they shall also have had more opportunity to develop an immuno-subversive armamentarium. The tumors that fail to develop such immuno-evasive mechanisms, which indeed may be a substantial proportion of them, possibly regress and are never detected clinically. The phenomenon of regressor tumors in mice [22], the increased incidence of cancers in immunologically suppressed patients [23], and the recent evidence affirming the role of immunological surveillance against tumors in mice [24], all indicate that this might indeed be the case. The phenomena of immune editing and immune evasion [25] must therefore be considered central to development of malignancy.

Our model is consistent with the fact that a number of true tumor-specific mutations have been identified in human and mouse tumors, and that these are individually tumor-specific [26], [27], [28], [29], [30], [31], [32], [33], [34], [35], [36], [37], [38], [39]. In fact, in almost every instance where immune response can be correlated to tumor rejection, the immune response is directed to these true tumor-specific mutations [26], [31], [33], [34]. However, only a relatively small number of individually specific mutations have been detected and structurally defined. We suggest that at least two reasons for this possible discrepancy; one, that the epitopes establish a hierarchy such that only the dominant epitopes are identified. Assarson et al [17]. estimate that 10 percent or fewer potential epitopes may be detected for this reason. Secondly, the methodological logistics of identifying epitopes of tumors are heavily biased towards detection of shared, and not true tumor-specific epitopes.

Our results have an important bearing on the immunogenicity of cancer stem cells. Regardless of the merits of the evidence supporting their existence [40], our results suggest that cancer stem cells shall harbor few mutations due to their asymmetric proliferative properties, and hence shall be inherently poorly immunogenic. They may also therefore be poorly responsive to immunotherapy.

### Implications for immunotherapy of human cancers

Our results suggest that human tumors of even clinically modest sizes harbor significant numbers of true tumor-specific epitopes generated as a result of the spontaneous mutations that are inalienably associated with cell division. These tumor-specific epitopes are predicted to be unique to each individual tumor because of the randomness of the mutation process. These considerations suggest a renewed emphasis on individualized immunotherapy of human cancer. Preliminary positive results from randomized Phase 3 clinical trials where autologous tumor-derived heat shock protein-peptide vaccines - which are based on the individually specific immunogenicity of cancers, are consistent with our model [41], [42]. The most extreme form of individualization of immunotherapy would of course consist of sequencing of the entire genome of each patient's tumor, followed by listing of the unique tumor-specific epitopes and immunization against a panel of such epitopes [43]. Our modeling predicts that the number of such epitopes shall not be inordinately large. With the rapid and continuing decline in the cost of sequencing, such approaches are not beyond the bounds of possibility in the near future. Further, considering that tumors already harbor a substantial immunogenic repertoire, a renewed effort towards dis-inhibition of immune responses, such as through blocking antibodies to CTLA4 [31], [44] or other such molecules, or through disruption of T regulatory networks [45], in combination with individualized vacci-therapy, may offer the best chance of success.

## References

[1] Simpson AJ (1997). The natural somatic mutation frequency and human carcinogenesis.. Adv Cancer Res.

[2] Beckman RA, Loeb LA (2005). Genetic instability in cancer: theory and experiment.. Semin Cancer Biol.

[3] Lengauer C, Kinzler KW, Vogelstein B (1998). Genetic instabilities in human cancers.. Nature.

[4] Fishel R, Lescoe MK, Rao MR, Copeland NG, Jenkins NA (1993). The human mutator gene homolog MSH2 and its association with hereditary nonpolyposis colon cancer.. Cell.

[5] Rajagopalan H, Nowak MA, Vogelstein B, Lengauer C (2003). The significance of unstable chromosomes in colorectal cancer.. Nat Rev Cancer.

[6] Tomlinson I, Sasieni P, Bodmer W (2002). Commentary: How Many Mutations in a Cancer?. Am J Path.

[7] Srivastava PK (1993). Peptide-binding heat shock proteins in the endoplasmic reticulum: role in immune response to cancer and in antigen presentation.. Adv Cancer Res.

[8] Sette A, Sidney J (1999). Nine major HLA class I supertypes account for the vast preponderance of HLA-A and-B polymorphism.. Immunogenetics.

[9] Sidney J, Peters B, Frahm N, Brander C, Sette A (2008). HLA class I supertypes: a revised and updated classification.. BMC Immunol.

[10] SYFPEITHI: database for MHC ligands and peptide motifs.. http://www.syfpeithi.de.

[11] Srivastava PK, Old LJ (1988). Individually distinct transplantation antigens of chemically induced mouse tumors.. Immunology Today.

[12] Basombrio MA Search for common antigenicities among twenty-five sarcomas induced by methylcholanthrene.. Cancer Research.

[13] International Human Genome Sequencing Consortium (2001). Initial sequencing and analysis of the human genome.. Nature.

[14] Segal NH, Parsons DW, Peggs KS, Velculescu V 2008 Epitope landscape in breast and colorectal cancer.. Cancer Research.

[15] Scanlan M, Stauffer Y, Theiler G, Zahn M, Jongeneel V (2004, updated 2006). CT gene database.. Cancer Immunity.

[16] van der Bruggen P, Stroobant V, Van Pel A, Van den Eynde B (2001, updated 2007). T cell defined tumor antigens.. Cancer Immunity.

[17] Assarsson E, Sidney J, Oseroff C, Pasquetto V, Bui HH (2007). A quantitative analysis of the variables affecting the repertoire of T cell specificities recognized after vaccinia virus infection.. J Immunol.

[18] Yewdell J (2006). Confronting complexity: real world immunodominance in CD8+ anti-viral T cell responses.. Immunity.

[19] Stoler D, Chen N, Basik M, Kahlenberg M, Rodriguez-Bigas M (1999). The onset and extent of genomic instability in sporadic colorectal tumor progression.. Proc Natl Acad Sci USA.

[20] Greenman (2007). Patterns of somatic mutation in human cancer genomes.. Nature.

[21] Gatenby RA, Frieden BR (2002). Application of information theory and extreme physical information to carcinogenesis.. Cancer Res.

[22] Urban JL, Schreiber H (1992). Tumor antigens.. Annu Rev Immunol.

[23] Andres A (2005). Cancer incidence after immunosuppressive treatment following kidney transplantation.. Crit Rev Oncol Hematol.

[24] Shankaran V, Ikeda H, Bruce AT, White JM, Swanson PE (2001). IFNgamma and lymphocytes prevent primary tumour development and shape tumour immunogenicity.. Nature.

[25] Dunn GP, Old LJ, Schreiber RD (2004). The three Es of cancer immunoediting.. Annu Rev Immunol.

[26] Baurain JF, Colau D, van Baren N, Landry C, Martelange V (2000). High Frequency of Autologous Anti-Melanoma CTL Directed Against an Antigen Generated by a Point Mutation in a New Helicase Gene.. J Immunol.

[27] Chiari R, Foury F, De Plaen E, Baurain J-F, Thonnard J (1999). Two Antigens Recognized by Autologous Cytolytic T Lymphocytes on a Melanoma Result from a Single Point Mutation in an Essential Housekeeping.. Gene Cancer Res.

[28] Coulie PG, Lehmann F, Lethe B, Herman J, Lurquin C (1995). A Mutated Intron Sequence Codes for an Antigenic Peptide Recognized by Cytolytic T Lymphocytes on a Human Melanoma.. Proceedings of the National Academy of Sciences.

[29] Dubey P, Hendrickson RC, Meredith SC, Siegel CT, Shabanowitz J (1997). The Immunodominant Antigen of an Ultraviolet-induced Regressor Tumor Is Generated by a Somatic Point Mutation in the DEAD Box Helicase p68.. J Exp Med.

[30] Echchakir H, Mami-Chouaib F, Vergnon I, Baurain J-F, Karanikas V (2001). A Point Mutation in the alpha-Actinin-4 Gene Generates an Antigenic Peptide Recognized by Autologous Cytolytic T Lymphocytes on a Human Lung Carcinoma.. Cancer Res.

[31] Hodi SF, Mihm MC, Soiffer RJ, Haluska FG, Butler M, Seiden MV (2003). Biologic activity of cytotoxic T lymphocyte-associated antigen 4 antibody blockade in previously vaccinated metastatic melanoma and ovarian carcinoma patients.. Proceedings of the National Academy of Sciences.

[32] Ikeda H, Ohta N, Furukawa K, Miyazaki H, Wang L (1997). Mutated mitogen-activated protein kinase: A tumor rejection antigen of mouse sarcoma.. Proceedings of the National Academy of Sciences.

[33] Karanikas V, Colau D, Baurain J-F, Chiari R, Thonnard J, Gutierrez-Roelens I (2001). High Frequency of Cytolytic T Lymphocytes Directed against a Tumor-specific Mutated Antigen Detectable with HLA Tetramers in the Blood of a Lung Carcinoma Patient with Long Survival.. Cancer Res.

[34] Lennerz V, Fatho M, Gentilini C, Frye RA, Lifke A (2005). The response of autologous T cells to a human melanoma is dominated by mutated neoantigens.. Proceedings of the National Academy of Sciences.

[35] Matsutake T, Srivastava PK (2001). The immunoprotective MHC II epitope of a chemically induced tumor harbors a unique mutation in a ribosomal protein.. Proceedings of the National Academy of Sciences.

[36] Monach PA, Meredith SC, Siegel CT, Schreiber H (1995). A unique tumor antigen produced by a single amino acid substitution.. Immunity.

[37] Sensi M, Nicolini G, Zanon M, Colombo C, Molla A (2005). Immunogenicity without Immunoselection: A Mutant but Functional Antioxidant Enzyme Retained in a Human Metastatic Melanoma and Targeted by CD8+ T Cells with a Memory Phenotype.. Cancer Res.

[38] Takenoyama M, Baurain J-F, Yasuda M, So T, Sugaya M (2006). A point mutation in the NFYC gene generates an antigenic peptide recognized by autologous cytolytic T lymphocytes on a human squamous cell lung carcinoma.. Int J Cancer.

[39] Zorn E, Hercend T (1999). A natural cytotoxic T cell response in a spontaneously regressing human melanoma targets a neoantigen resulting from a somatic point mutation.. Eur J Immunol.

[40] Lobo NA, Shimono Y, Qian D, Clarke MF (2007). The biology of cancer stem cells.. Annu Rev Cell Dev Biol.

[41] Srivastava PK (1996). Do human cancers express shared protective antigens? or the necessity of remembrance of things past.. Semin Immunol.

[42] Testori A, Richards J, Whitman E, Mann B, Lutzky J, Camacho L, Parmiani G, Hoos A, Yuh L, Gupta R, Srivastava PK (2008). Comparison of tumor-derived heat shock protein gp96-peptide complex vaccine (Vitespen) and Physician's choice in a Randomized Phase 3 trial in patients with Stage IV melanoma.. J Clin Oncology.

[43] Rammensee HG, Weinschenk T, Gouttefangeas C, Stevanovic S (2002). Towards patient-specific tumor antigen selection for vaccination.. Immunol Rev.

[44] Peggs KS, Quezada SA, Korman AJ, Allison JP (2006). Principles and use of anti-CTLA4 antibody in human cancer immunotherapy.. Curr Opin Immunol.

[45] Kretschmer K, Apostolou I, Jaeckel E, Khazaie K, von Boehmer H (2006). Making regulatory T cells with defined antigen specificity: role in autoimmunity and cancer.. Immunol Rev.

